# Global Comparison of Changes in the Number of Test-Positive Cases and Deaths by Coronavirus Infection (COVID-19) in the World

**DOI:** 10.3390/jcm9061904

**Published:** 2020-06-18

**Authors:** Akihiro Hisaka, Hideki Yoshioka, Hiroto Hatakeyama, Hiromi Sato, Yoshihiro Onouchi, Naohiko Anzai

**Affiliations:** 1 Clinical Pharmacology and Pharmacometrics, Graduate School of Pharmaceutical Sciences, Chiba University, 1-8-1, Inohana, Chuo-ku, Chiba 260-8675, Japan; ysokhdk@gmail.com (H.Y.); h-hatakeyama@chiba-u.jp (H.H.); hiromi-s@chiba-u.jp (H.S.); 2 Department of Public Health, Graduate School of Medicine, Chiba University, Chiba, 1-8-1, Inohana, Chuo-ku, Chiba 260-8670, Japan; onouchy@chiba-u.jp; 3 Department of Pharmacology, Graduate School of Medicine, Chiba University, Chiba, 1-8-1, Inohana, Chuo-ku, Chiba 260-8670, Japan; anzai@chiba-u.jp

**Keywords:** COVID-19, coronavirus, infectious disease, infection management, PCR test, mortality, kinetic analysis

## Abstract

Global differences in changes in the numbers of population-adjusted daily test-positive cases (NPDP) and deaths (NPDD) by COVID-19 were analyzed for 49 countries, including developed and developing countries. The changes as a proportion of national population were compared, adjusting by the beginning of test-positive cases increase (BPI) or deaths increase (BDI). Remarkable regional differences of more than 100-fold in NPDP and NPDD were observed. The trajectories of NPDD after BDI increased exponentially within 20 days in most countries. Machine learning analysis suggested that NPDD on 30 days after BDI was the highest in developed Western countries (1180 persons per hundred million), followed by countries in the Middle East (128), Latin America (97), and Asia (7). Furthermore, in Western countries with positive rates of the PCR test of less than 7.0%, the increase in NPDP was slowing-down two weeks after BPI, and subsequent NPDD was only 15% compared with those with higher positive rates, which suggested that the situation of testing might have affected the velocity of COVID-19 spread. The causes behind remarkable differences between regions possibly include genetic factors of inhabitants because distributions of the race and of the observed infection increasing rates were in good agreement globally.

## 1. Introduction

The novel coronavirus diseases 2019 (COVID-19) has been spreading globally and was declared as a pandemic by the WHO on 11 March 2020 [1]. Following the outbreak in Wuhan, China, in December 2019 [2], the number of test-positive cases in Italy, the United States, Spain, France, and Germany has surpassed the number of Chinese cases as of early April 2020, and more countries will soon follow. Nations and cities in which outbreaks have occurred have adopted strong measures such as overall shutdowns. However, the number of victims is growing rapidly. The cumulative number of test-positive cases worldwide reached 1,619,964 on 10 April 2020 [3] and is increasing at a daily rate of approximately 80,000 new cases. As of early April, the cumulative number of cases in Asia is around 270,000, which accounts for 16.5% of the world’s cases. The number of cases in Europe and North America is higher than in Asia, accounting for 48.8% and 30.9% of the world’s cases, respectively. The densities of cumulative test-positive cases are 18.4- and 14.5-fold higher in Europe and North America than in Asia, respectively, and the difference is constantly increasing. In contrast, the density of test-positive cases in Africa is very low, at only 0.17-fold that of Asia. The number of test-positive cases is ever-increasing and depends on the timing of the outbreak, and differences in medical environments and policies between countries. Thus, it is difficult to interpret simple differences in the number of cases between continents. However, large biases between different continents are a fact and serious considerations must be given to how to reasonably halt the infections of different degrees depending on the continental, and at the same time, the possibility of spreading to areas where the infection is not yet widespread [4].

In contrast, the number of test-positive cases may be affected by the number of conducted PCR tests. The PCR test is now widely accepted as the gold standard method for confirming COVID-19 [5]. Not quarantining infected people may increase the risk of an explosive epidemic. Countries that actively conduct PCR tests believe that testing is indispensable to identify infected individuals and quarantine them. These countries tested anyone showing COVID-19 symptoms. However, even in countries where the infection has widely spread, it is difficult to test everyone because the PCR test is rather laborious and the infection rate in such countries is only approximately 0.1%. In addition, even if a virus is present in the body, false negatives may occur [6]; therefore, it is difficult to ensure that every infected individual is quarantined. Conversely, some countries seem to fear that aggressive test regimes may waste resources, and are unenthusiastic to perform widespread PCR testing. Those countries tested only people who both (a) have symptoms and (b) met specific criteria, such as admission to hospital, contact with a known case, or return from overseas. Different countries have implemented different testing policies and possess different testing capacities. Aiming to investigate the current state of the COVID-19 pandemic by analyzing the number of test-positive cases alone may lead to bias.

This study aimed to investigate regional differences in the global COVID-19 outbreak by analyzing the number of deaths, which is a more direct consequence of the infection, in addition to the number of test-positive cases in various countries. The countries were selected simply by using the top 49 countries in terms of number of test-positives as of the end of March 2020, thus including both developed and developing countries. Regarding mortality, there is an option to analyze excess mortalities (differential deaths from historical average) because the announced mortality of COVID-19 has been possibly affected by differences in policies, the epidemic situations, and the ability to detect mortality. However, excess mortalities are available only in limited countries. In addition, excess mortalities may be affected by changes in deaths due to other illnesses and traffic accidents. Therefore, in this study, the number of deaths due to COVID-19 announced by each country was analyzed. However, the reliability of the mortality data needs to be interpreted with caution in countries where the COVID-19 epidemic had a serious impact on social infrastructure. 

Generally, the basic reproduction number (*R*_0_) is preferred to assess the risk of infectious diseases including COVID-19 [7]. However, estimating *R*_0_ requires an estimation of the number of infected individuals, infection rate, quarantine rate, and recovery rate, and these parameters would be potentially time-dependent, non-linear, and diverse among populations. Moreover, the reliability of the estimated *R_0_* is difficult to verify, owing to the uncertainty of the information used for the calculation. Thus, it is not easy to compare *R*_0_ among different countries in a timely manner. For this reason, we focused our analysis on changes in the number of test-positive cases and deaths adjusted by the population of each country and considering the timing of the outbreak.

## 2. Materials and Methods

According to the Kermac–McKendrick model [8], the number of infected subjects at time *t*, normalized to the total susceptibility population, *I(t)*, obeys the following Malthus’ law:

*I(t) = I(0) e^(β-γ)t^,* (1)

where *β* is the infection rate and *γ* is the recovery and isolation rate, respectively. Here, infected subjects include those who are asymptomatic. Immediately after the initiation of the outbreak, which is typically caused by the infection being brought in from abroad, *γ = 0* because neither recovery nor isolation is possible. Currently, in most countries, the PCR testing starts when individuals with symptoms of infection appear, so the period during which *γ* is 0 continues until the symptoms become apparent after infection occurs. We call this “the hidden phase”. After detection, the isolation rate increases with intensive testing, and the number of test-positive cases increases explosively. We call this “the explosive increase phase”. Thereafter, the quarantine rate increases and is tentatively kept at a constant value depending on the testing system of each country. Some asymptomatic people may recover during this period, which also raises *γ*. The ratio of the number of test-positive cases to infected cases becomes approximately constant due to the equilibrium of these rates. Thus, an increase due to Malthus’ law is observed, and we call it “the exponential increase phase”. From several days to about two weeks later, due to social responses to the infection [9], *β* gradually decreases. Simultaneously, deaths and recovered individuals appear mostly from quarantined or hospitalized people. We call this “the deceleration phase”. As the deceleration phase continues, *β* becomes smaller than *γ*, and the number of infected people begins to decrease, and later the epidemic ends. 

It should be noted that the number of test-positive cases analyzed in this study was smaller than the number of infected people and was observed only after the hidden phase. The ratio of test-positive cases to infected cases should be time-dependent especially in the initial period. The number of deaths was observed after the deceleration phase. The basic reproduction number is represented by *R_0_ = β/γ*, but its meaning is different depending on the phase. To estimate *R_0_*, it is necessary to select an appropriate timing and to estimate the detection ratio and recovery rate in each country; and thus, this was not analyzed in this study.

Daily numbers of test-positive cases and deaths by COVID-19 reported from all countries worldwide were downloaded from the webpage of the European Center for Disease Prevention and Control (ECDC) [10] as of 8 April 2020. The analysis was narrowed down to the top 50 infected countries as of 29 March. Because the Princess Diamond cruise ship was excluded, the actual analysis was performed for 49 countries. The number of population-adjusted daily test-positive cases (NPDP) and the number of population-adjusted daily deaths (NPDD) were calculated as five-day moving average for a population of hundred million. The first and last points were calculated as a three-day moving average. The beginning of test-positive cases increase (BPI) was defined as a point at which the average NPDP for two consecutive days exceeded 10. The beginning of deaths increase (BDI) was defined as a point at which the average NPDD for two consecutive days exceeded 1 for Asian countries and 5 for non-Asian countries, respectively. Vietnam, Russia, and South America did not meet the criteria for BDI. Due to a small population, the BDI of Singapore, Luxembourg, and Iceland was not reliable and thus these countries were excluded from the BDI related analysis. All the numerical data is provided as Supplementary Material 1. The virus testing statistics by country were obtained from the English version of Wikipedia (Supplementary Material 2) [11]. The described information is based on the official announcement of each country, and its sources are all listed.

Regression analysis was performed on the trajectories of NPDP after BPI and NPDD after BDI using the gradient boosting decision tree algorithm [12]. A dummy covariate representing a global region or a range of the positive rate of the PCR test was used. The analysis was performed using GradientBoostingRegressor function of the scikit-learn library (version 0.22.2) [13,14] on Python (version 3.7.3) [15]. The 95% confidence interval was calculated by repeating the country-level bootstrap analysis of 1000 times. The data and sample code of machine learning analysis are provided as Supplementary Materials 3–5.

## 3. Results

**Figure 1:**
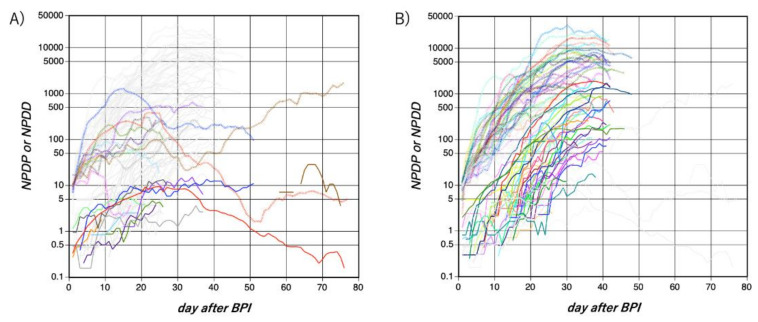
Time courses of NPDP and NPDD after BPI by novel coronavirus diseases 2019 (COVID-19) in Asian countries (except for the Middle East. Panel (**A**) and non-Asian countries (Panel (**B**)). The upper dotted lines and lower solid lines represent NPDP and NPDD, respectively. The colors of NPDP and NPDD correspond to each country. Lines in very light gray indicate lines in non-Asian and Asian countries in Panels A and B, respectively, for comparison. Refer to Figure 2 and Figure 3 to identify each country. NPDP: number of population-adjusted daily test-positive cases. NPDD: number of population-adjusted daily deaths. BPI: beginning of the test-positive cases increase.

Figure 1 shows changes in NPDP and NPDD per 100 million in each country from the BPI. Most countries showed a rapid increase in NPDP for about 5 days after the BPI, which was probably the explosive increase phase. From 5–20 days after BPI, an exponential increase was observed in many countries. Asian (excluding the Middle East, the same applies hereinafter) and non-Asian countries (Europe, Middle East, Americas, Africa, and Oceania, the same applies hereinafter) showed very different trajectories later than 20 days after BPI, that is, the deceleration phase. In Asian countries, NPDP fluctuated mostly in the range of 10 to 1000. In some countries, the fluctuation has continued for more than 70 days. In most non-Asian countries, however, NPDP increased steeply and then reached a plateau of 1000 or more by 30 days after BPI. In some European countries, NPDP reached almost 10,000. A plateau of NPDP does not indicate a plateau in the number of cumulative test-positive cases, rather its linear increase. The NPDP analysis found no noticeable regional differences between Europe, Africa, the Americas, and the Middle East. The outbreak is still in the very early phase in the South Asian countries, i.e., Pakistan and India, but it seems that their NPDP time courses are more like the East Asian countries than the European countries currently. Fortunately, the NPDPs in Africa and in Oceania (Australia and South Africa, respectively) have recently entered a decreasing phase, although only one country in each of these areas was analyzed.

In the change in NPDD, unlike NPDP, almost no initial explosive increase was observed. The increase in NPDD in Asian countries was modest, with values of about 10 or less on the 20th day after BPI. In some countries, NPDD was less than 1. In contrast, in most non-Asian countries, NPDDs rose significantly, as high as 50–2000 at 30 days after BPI. However, there are still many countries where the period after BPI is still short, and the value is not yet definitive. In many countries, NPDD also reached a plateau at 30 days after BPI, and the cumulative number of deaths had shifted from an exponential increase to a linear increase.

**Figure 2:**
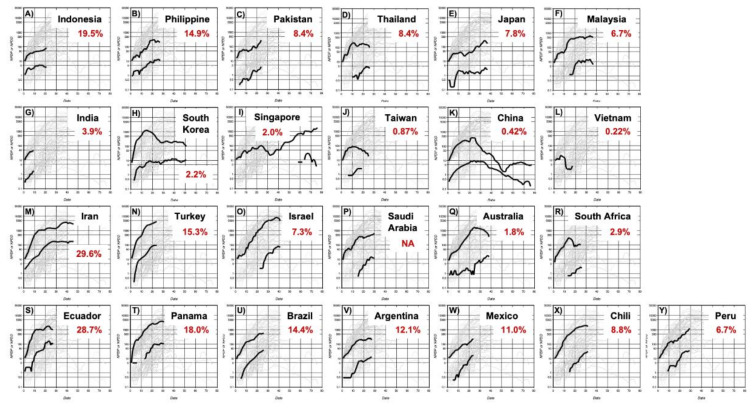
Time courses of NPDP and NPDD by COVID-19 after BPI in Asian countries (Panels (**A**–**L**)), the Middle East (Panels (**M**–**P**)), Oceania (Panel (**Q**)), South Africa (Panel (**R**)), and Latin America (Panels (**S**–**Y**). Numbers in red represent the positive rate of the PCR test. The countries are placed in decreasing order of the positive rate in each region. The upper and lower bold lines in each panel are NPDP and NPDD, respectively, except for Vietnam where no death has been recorded. The scales in all panels are the same as those in Figure 1. *: Positive rate in Guangdong. NA: Not available. NPDP: number of population-adjusted daily test-positive cases. NPDD: number of population-adjusted daily deaths. BPI: beginning of the test-positive cases increase.

**Figure 3:**
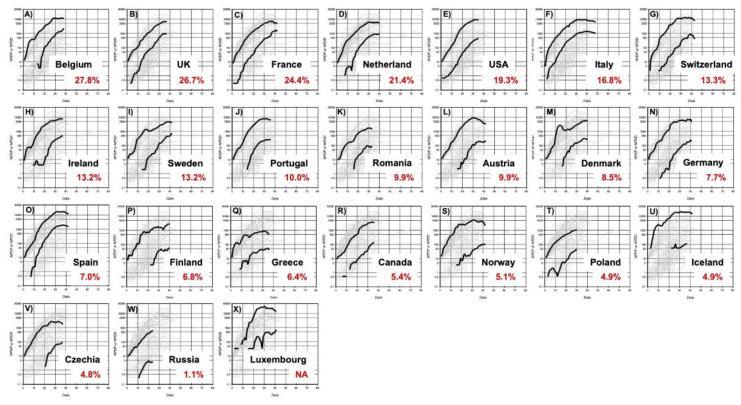
Time courses of NPDP and NPDD after BPI by COVID-19 in countries in Europe and North America (Panels (**A**–**X)**). Numbers in red represent the positive rate of the PCR test. The countries are placed in decreasing order of positive rate. The upper and lower bold lines in each panel are NPDP and NPDD, respectively. The scales in all panels are the same as those in Figure 1. NA: Not available. NPDP: number of population-adjusted daily test-positive cases. NPDD: number of population-adjusted daily deaths. BPI: beginning of the test-positive cases increase.

Figure 2 and Figure 3 show individual NPDD and NPDP profiles for countries ordered by the positive rate of the PCR tests. In Asian countries, the relationship between the spread of the infection and the positive rate was not clear. Nevertheless, NPDDs were very low in countries with the lowest positive rates such as Taiwan, China, and Vietnam. A similar tendency was also observed in the countries in the Middle East and Latin America. In Western countries, it was apparent that higher NPDD occurred in the countries with a higher positive rate and shorter lag time.

**Figure 4:**
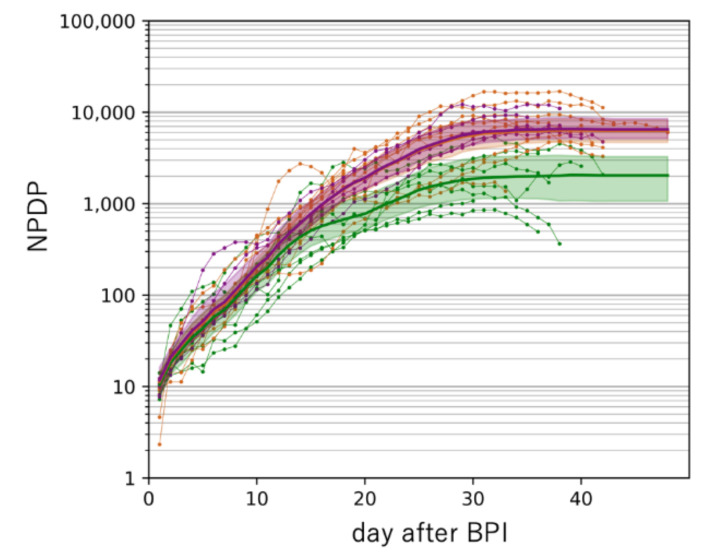
Estimation of the time course of NPDP after BPI in Western countries by machine learning analysis classified by the positive rate of the PCR test. Green, orange, and purple lines indicate positive rates of 0.0–6.9%, 7.0–16.9%, and 17.0–28.0%, respectively. Each colored area represents a 5–95% confidence interval of the median estimated by bootstrap analysis. The orange and purple lines and areas completely overlapped. NPDP: number of population-adjusted daily test-positive cases. BPI: beginning of the test-positive cases increase. PCR: Polymerase chain reaction.

Figure 4 shows the results of machine learning analysis on the trajectory of NPDP after BPI, classified by the PCR-positive rates. The thresholds of 7.0% and 17.0% were adopted as values close to the quartile. There was no difference in the degree of increase in NPDP up to two weeks after BPI between countries with low and high positive rates. However, at two weeks after BPI, the slope of change in NPDP was attenuated only in countries with a positive rate of less than 7.0%. Subsequently, the difference in NPDP between the country groups (countries with <7.0% and those with >7.0% of positive rates) expanded to approximately 3-fold at 30 days after BPI.

**Figure 5:**
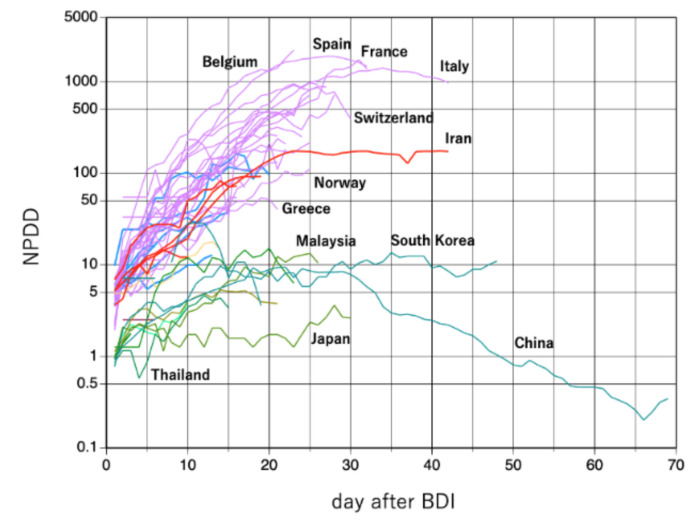
Time courses of NPDD after BDI by COVID-19. Green, red, blue, and purple lines represent Asia, the Middle East, Latin America, and Western countries, respectively. Some noticeable countries are labeled but refer to Figure 2 and Figure 3 to identify each country. NPDD: number of population-adjusted daily deaths. BPI: beginning of the test-positive cases increase.

There would be no extreme difference in the period between the timing of infection and death in countries where medical care has reached a certain level. Therefore, if NPDD increased immediately after the NPDP increase, the true infection should have occurred somewhat earlier than was detected by the increase in NPDP [16]; that is, “the hidden phase” should have been longer. For this reason, we also analyzed changes in NPDD after BDI (Figure 5). The trajectories of NPDD after BDI were consistent for all the countries tested. NPDD showed an exponential increase until approximately 20 days after BDI and then reached a plateau by 25 days. It is encouraging that the peaks of NPDD for Italy and Spain seem to have passed. At present, only China has shown a clear decline in NPDD. By this analysis, it became somewhat apparent that NPDD trajectories of countries in the Middle East and Latin America follow an intermediate transition between Asian and Western countries.

**Figure 6:**
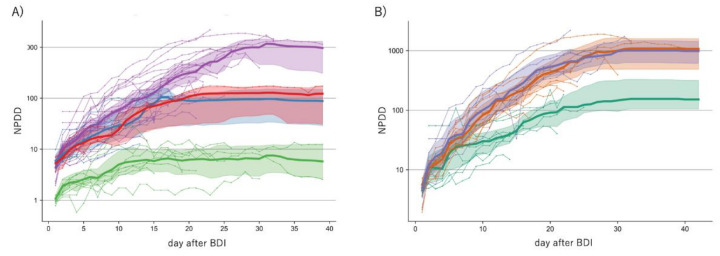
Estimation of the time course of NPDD after BDI by machine learning analysis classified by the global region (Panel (**A**)) and by the positive rate of the PCR test (Panel (**B**)). In Panel (**A**), yellow-green, red, blue, and purple lines indicate countries in Asia (excluding the Middle East), the Middle East, Latin America, and Western (Europe, Oceania, and North America), respectively. In Panel (**B**), only Western countries were analyzed. Green, orange, and blue lines indicate the positive rates of 0.0–6.9%, 7.0–16.9%, and 17.0–28.0%, respectively. Each colored area represents a 5–95% confidence interval of the median estimated by bootstrap analysis. NPDD: number of population-adjusted daily deaths. BPI: beginning of the test-positive cases increase.

Figure 6 shows the results of machine learning analysis on the transition of NPDD after BDI. Due to differences in BDI criteria between Asian and non-Asian countries, BDI in non-Asian countries is approximated to be a few days earlier than the currently observed value. Even though such a difference was taken into consideration, distinct global regional differences were demonstrated by the analysis because the 95% confidence intervals (CI) did not overlap between Asian and non-Asian countries (Figure 6A). The analysis estimated that the medians of NPDD 30 days after BDI were 1180, 128, 97, and 7 in Western countries, the Middle East, Latin America, and Asia, respectively. Africa was not analyzed because only one country was included. The NPDDs in Western countries was classified by the PCR-positive rates and analyzed by using machine learning (Figure 6B). In countries with a positive rate of less than 7%, the number of deaths after 30 days after BDI was expected to be 15% in countries with a positive rate of equal or more than 17.0%. No difference in NPDD was detected between countries with a positive rate of 7.0–16.9% and 17.0–28.0%. The positive rate was the lowest (1.8%) in Australia, which has successfully suppressed deaths to the lowest level in Western countries at the time of this analysis.

## 4. Discussion

In this study, remarkable regional differences in changes in NPDP and NPDD became apparent between Asian countries (excluding the Middle East) and non-Asian countries (Figure 1). In NPDDs of countries in the Middle East and Latin America, small differences were also found between the Asian and Western countries (Figure 5 and Figure 6). COVID-19 outbreaks occurred everywhere on Earth, which kinetically means that *R*_0_ is considerably higher than 1.0 in any country without precautionary measures and efficient quarantine. However, once appropriate social measures were undertaken, considerable regional differences appeared from 20 days after the outbreak. Between-country differences and fluctuations in NPDP in Asia may be due to accidental outbreaks or intermittent inputs of infected people from overseas. Attention should be paid to recent rises in the NPDPs in several Asian countries, such as China and Singapore, where NPDP has been well controlled for a while. Infection with different lineages of SARS-Cov-2 [17] should need to be considered because some small outbreaks in Asian countries synchronized with outbreaks in Europe. Nevertheless, it seems that the later outbreaks in Asian countries were smaller than the first ones.

In contrast, non-Asian countries showed steep increasing trajectories for both NPDP and NPDD. In particular, in some European countries where the serious outbreaks occurred, NPDP and NPDD reached very high numbers, approximately 10,000 and 1000, respectively, by 30 days after BPI. However, at the same time, it should be noted that NPDP has recently entered the decline phase in some European countries such as France, Italy, Switzerland, Portugal, Spain, Greece, Norway, and Austria (Figure 3). In China, the peak of NPDP occurred on February 6 and, given the recent partial unblocking of Wuhan, the containment in June or July would not be impossible in Europe.

The reason behind the reduced spread of the virus in Japan, compared to Europe and other Western countries, has been discussed. It was suspected that the smaller number of PCR tests carried out in Japan is responsible for this [18]. The results of this analysis showed that Japan, as well as all Asian countries, had considerably slower spread of infection compared with the European countries when test-positive cases were adjusted for population size. In addition, similar regional differences were observed, not only in NPDP but also in NPDD, indicating that the difference in the number of tests did not significantly affect the evaluation of the differences in the rate of infection between Asian and non-Asian countries. In addition, it should be noted that the positive rate of the PCR test in Asian countries is generally not higher than that in Western countries for the sake of a smaller number of test-positive cases (Figure 2 and Figure 3).

Differences in the spread of infection classified by a positive rate for the PCR tests were compared within Western countries where the timing and situation of spread of COVID-19 were similar. The increase in NPDP during 2 weeks after BPI was similar in all analyzed countries, whereas in countries where the positive rate was below 7.0%, the velocity of increase in NPDP decreased thereafter (Figure 4). COVID-19 symptoms often develop approximately 2 weeks after infection, and thus a slowing-down of the infection spread 2 weeks after BPI seems reasonable as a consequence of the aggressive PCR testing. However, confounding factors, such as governmental policies, the medical environment, welfare systems, socio-behavioral factors, and the age and ethnic composition of the people, may also need to be considered. On the other hand, since many countries with low or high positive rates are classified generally as high-income, it was suggested that the other factors had a stronger influence than income as covariates affecting the spread of infection. The relationship between test aggressiveness and consequences of an infection is an important perspective, perhaps not previously considered in other infectious disease outbreaks.

The estimated NPDD at 30 days after BDI in Western countries with a positive rate of the PCR test of less than 7.0% was only 15% compared to countries with a higher positive rate (Figure 6B). In this study, it is difficult to define the target positive rate for effective attenuation of deaths, however, 7.0% is probably not enough because some countries with a positive rate of less than 2%, such as Australia and Russia, NPDDs were effectively suppressed further (Figure 3). Moreover, in Asia, deaths by COVID-19 have been almost successfully contained currently in China and Thailand where the positive rate was less than 1% (Figure 2). The causal relationships between the testing policy and the positive rate, and between the positive rate and spread of infection need to be verified thoroughly in the future.

It is unclear whether the large interregional differences in responses to the COVID-19 observed in this study were due to genetic differences between the inhabitants. Ethnic differences in response to infectious diseases have rarely been reported. However, this possibility cannot be denied because distributions of the race and of observed infection increasing rates were in good agreement globally. Inhabitants in East Asia and Southeast Asia are Mongoloids, mostly of native descent, whereas Western countries are more heterogenic and predominately by Caucasians. The Middle East, Pakistan, and India are classified generally as Caucasians, but they differ considerably from Europeans. In this analysis, the results from Pakistan and India were closer to those of Asian countries, while Iran had an intermediate increasing infection rate between Asian and non-Asian countries. Although the Middle East and Latin America were classified as an independent group from Europe and other parts of Asia (Figure 6A), the period after BDI is not yet sufficient to conclusively determine if countries in these regions are different from Europe or Asia. Those of African descent do not account for the majority of the population of any nation in this analysis, and it is not yet possible to predict whether the infection will spread in Africa at the same rate as in Europe. In multiethnic countries, such as the United States, the racial background of the patients needs to be analyzed carefully. However, such information is not yet available.

The large interregional differences in the spread of COVID-19 analyzed in this study might be related to the frequency of individuals who are genetically susceptible or resistant to COVID-19 in each country. With regard to HIV transmission, C-C chemokine receptor 5 (CCR5) serves as the predominant co-receptor for viral entry during the initial transmission, and it is established that a homozygous Δ32 mutation which prevents its expression on the cell surface confers resistance to the infection [19]. It would be possible that an analogous mechanism works also in the case of SARS-Cov-2. SARS coronaviruses utilize ACE2 as an essential receptor for cell fusion for infection [20]. *ACE2,* encoded on Xp22 at the location where genes are known to escape X-inactivation, may contribute to the phenotypic differences between sexes and the tissue-specific differences in X-inactivation [21]. An intronic variant of *ACE2* (rs2285666, also known as G8790A) has been associated with hypertension [22]. Wu et al., reported that the serum ACE2 level in individuals with an AA genotype was 1.5-fold higher [23]. According to the 1000 Genomes project, the allele frequency of A was the lowest in African (21.1%) and European populations (23.5%), and the highest in East Asian (53.7%). The frequencies in South Asian (47.9%) and Latin American (33.6%) are intermediates. It is unknown whether ACE2 expressions in the target tissues of infection correlate with that in the serum. If the A allele or other linked variant alleles have a protective effect against SARS-Cov-2, the different allele frequencies among ethnicities might be relevant to the regional differences in COVID-19 epidemiology observed in this study. Future research in this area is necessary.

Genetic variants could also modulate patients’ manifestation of COVID-19, and it might relate to the ethnic differences. The homozygotes of the C allele at rs12979860 (C/T) near the *IL28B* gene responded twice as well in sustained virologic response (SVR) to peginterferon (pegIFN) and ribavirin (RBV) therapy for chronic hepatitis C and B than the heterozygotes [24]. East Asians responded better than European Africans and the C allele frequency in East Asian (92.0%) is higher than those of European (69.1%) and African (33.1%). On the other hand, it has been reported that rs4042 in the *CXCL1* gene is related to the levels of some chemokines in the blood [25], which may suppress the progression of fibrosis in hepatitis C [26]. The frequency of the favorable A allele is higher in the order of African, Asian, and Caucasian populations [27]. Thus, the disease may progress faster in Caucasians than other ethnicities. Involvement of these variant alleles in the manifestation of COVID-19 is currently unknown.

It has been hypothesized that regional differences in COVID-19 impact could be partially explained by different national policies such as the Bacillus Calmette-Guérin (BCG) childhood vaccination [28]. In general, vaccines exert immune responses specific to a targeted pathogen by producing antibodies. However, it has been reported that BCG vaccination induces genome-wide epigenetic reprograming of human monocytes which is accompanied by significantly altered responses of innate immune cells [29]. The “trained immunity” from the BCG vaccination could protect against a non-related viral infection with an attenuated yellow fever virus vaccine strain by IL-1β-mediated responses. Higher IL-1β levels are also highly relevant to the direct protective effect against viral infection, such as the herpes simplex virus [30]. Even though further investigations are necessary, these observations could support the hypothesis that BCG may contribute to regional differences in COVID-19.

## 5. Conclusions

In this study, we showed that the number of population-adjusted cumulative deaths due to COVID-19 increased exponentially for about 20 days from the BDI, and then increased linearly after 25 days, in most countries in the world. Approximately 30 days after the BDI, remarkable regional differences in mortalities were found between Asia, the Middle East, Latin America, and Western countries. This regional difference may be affected by governmental policies, the medical environment, welfare systems (including BCG vaccination), and socio-behavioral factors, and the age composition of the people in each country. However, it is possible that host genetic factors influence individuals’ susceptibility, response to treatment and prognosis in SARS-Cov-2 infection, and investigation of these factors is an urgent issue for more efficient medical care and public health policymaking. In the future, it will be very important to quantitatively explore the factors that affect the rate of COVID-19 by further analyzing country-specific information. Although the change in the number of test-positive individuals is an important indicator of the rate of spread of infection, a multiple-fold discrepancy with the number of deaths exists, and thus careful estimation of the number of infected subjects is warranted. Extensive PCR testing carries the risk of causing social disruption by magnifying the initial explosive increase, but our analysis suggested that it might be effective for reducing the number of deaths. Further studies are necessary to verify our hypothesis. The machine learning analysis presented here is still preliminary; however, as the search for infection spreading factors progresses, prediction accuracy will be improved further, and it is expected to be useful for devising a rational strategy to terminate COVID-19.

## Supplementary Materials

The following are available online at https://www.mdpi.com/2077-0383/9/6/1904/s1 (readme.rtf, 1_AnalysisOfCOVID-19.xlsx, 2_PCRtest.xls, 3_data.csv, 4_sample_script.ipynb, and 5_sample_script.ipynb.pdf).
