# Supplementary material for: Global Comparison of Changes in the Number of Test-Positive Cases and Deaths by Coronavirus Infection (COVID-19) in the World
Source: J Clin Med. 2020 Jun 18;9(6):1904. doi: 10.3390/jcm9061904 (PMC7356890; doi:10.3390/jcm9061904)
Supplement: Supplementary file 1 [file jcm-09-01904-s001.zip › sup/readme.rtf]

1_AnalysisOfCOVID-19.xlsxRaw data for the study. The file includes ...· the number of test-positives and deaths by country, downloaded from ECDC (https://www.ecdc.europa.eu/en)2_PCRtest.xlsxInformation on virus testing by country, obtained from https://en.wikipedia.org/wiki/COVID-19_testing3_data.csvPreprocessed data for machine-learning analysis.4_sample_script.ipynbPython code for machine-learning analysis (jupyeter notebook file). The code reproduces a series of analysis the effects of the PCR test, including reading the data, optimizing a gradient boosting model with grid search, and graphing the prediction results with bootstrap confidence intervals.5_sample_script.ipynb.pdfPrintout of the execution result of "sample_script.ipynb".
