# Supplementary material for: Global Comparison of Changes in the Number of Test-Positive Cases and Deaths by Coronavirus Infection (COVID-19) in the World
Source: J Clin Med. 2020 Jun 18;9(6):1904. doi: 10.3390/jcm9061904 (PMC7356890; doi:10.3390/jcm9061904)

In [1]:
```python
import pandas as pd
import numpy as np

from datetime import datetime, timedelta

from matplotlib import pyplot as plt

from sklearn.ensemble import GradientBoostingRegressor
from sklearn.model_selection import GridSearchCV
```

In [2]:
```python
"""
NPDD: population-adjusted daily deaths
PCRpositive_digitized=0: PCRpositive, 0.00-6.90
PCRpositive_digitized=1: PCRpositive, 7.00-16.9
PCRpositive_digitized=2: PCRpositive, 17.0-
(https://en.wikipedia.org/wiki/COVID-19_testing)
"""
data = pd.read_csv('data.csv')
data.head(5)
```

Out[2]:

	iso-code	country	day	log10(NPDD)	lagtime	region	PCRtests	PCRpositive	PCRpositive_digi
**0**	410	Korea	1	-0.109579	3	asia	477304	2.2	
**1**	410	Korea	2	0.288249	3	asia	477304	2.2	
**2**	410	Korea	3	0.434249	3	asia	477304	2.2	
**3**	410	Korea	4	0.492341	3	asia	477304	2.2	
**4**	410	Korea	5	0.589167	3	asia	477304	2.2	

Ex.) Effects of PCR Test Positive Ratio

In [3]:
```python
feature_names = ['day', 'PCRpositive_digitized']
X_train, y_train = (data[data['region'] == 'western'][feature_names],
                    data[data['region'] == 'western']['log10(NPDD)'])

# define model
model = GradientBoostingRegressor(
    alpha=0.9, ccp_alpha=0.0, criterion='friedman_mse',
    init=None, learning_rate=0.1, loss='ls', max_depth=5,
    max_features=1, max_leaf_nodes=None,
    min_impurity_decrease=0.0,
    min_samples_leaf=1, min_samples_split=2,
    min_weight_fraction_leaf=0.2,
    n_estimators=9999, n_iter_no_change=10,
    random_state=42, subsample=0.2, tol=0.0001,
    validation_fraction=0.1, verbose=False, warm_start=False)

# optimize hyper-parameters by gridsearch
param_grid = {'max_depth': [3, 4, 5, ],
              'subsample': [0.8, 1, 0.5],
              'min_weight_fraction_leaf': [0., 0.1, 0.2, ]}
grid = GridSearchCV(model, param_grid, cv=5, verbose=0, n_jobs=-1,
                    return_train_score=True, refit=True)
grid.fit(X_train, y_train,)
cv_results = pd.DataFrame(grid.cv_results_).sort_values('rank_test_sco
re')

print('Best Params:', grid.best_params_)
print('mean_train_score', cv_results['mean_train_score'].iloc[0])
print('mean_test_score', cv_results['mean_test_score'].iloc[0])
```

```
Best Params: {'max_depth': 3, 'min_weight_fraction_leaf': 0.0, 'subs
ample': 0.5}
mean_train_score 0.8905797581702372
mean_test_score 0.8358382298832545
```

In [4]:
```python
# bootstrapping
X_boot = pd.concat([
    pd.DataFrame({
        'day': sorted(X_train['day'].unique()),
        'PCRpositive_digitized': 0,
    }),
    pd.DataFrame({
        'day': sorted(X_train['day'].unique()),
        'PCRpositive_digitized': 1,
    }),
    pd.DataFrame({
        'day': sorted(X_train['day'].unique()),
        'PCRpositive_digitized': 2,
    }),
])

y_boot = []
indices_shuffled = np.random.choice(X_train.index.unique(),
                                    size=(1000, len(X_train.index.uniq
ue())))
for _, index in enumerate(indices_shuffled):
    model_ = model.fit(X_train.loc[index], y_train.loc[index])
    y_boot.append(model_.predict(X_boot,))

y_boot = np.array(y_boot)

# visualize model predictions with bootstrap 95%CI
y_boot_0 = y_boot[:, X_boot['PCRpositive_digitized'] == 0]
y_boot_1 = y_boot[:, X_boot['PCRpositive_digitized'] == 1]
y_boot_2 = y_boot[:, X_boot['PCRpositive_digitized'] == 2]
plt.plot(np.quantile(y_boot_0, 0.5, axis=0), 'g-', label='positive < 7
%')
plt.plot(np.quantile(y_boot_0, 0.025, axis=0), 'g--')
plt.plot(np.quantile(y_boot_0, 0.975, axis=0), 'g--')
plt.plot(np.quantile(y_boot_1, 0.5, axis=0), 'b-', label='positive >=7
% positive <17%')
plt.plot(np.quantile(y_boot_1, 0.025, axis=0), 'b--')
plt.plot(np.quantile(y_boot_1, 0.975, axis=0), 'b--')
plt.plot(np.quantile(y_boot_2, 0.5, axis=0), 'r-', label='positive >=1
7%')
plt.plot(np.quantile(y_boot_2, 0.025, axis=0), 'r--')
plt.plot(np.quantile(y_boot_2, 0.975, axis=0), 'r--')
plt.legend()
```

Out[4]:
```
<matplotlib.legend.Legend at 0x11b2b1910>
```

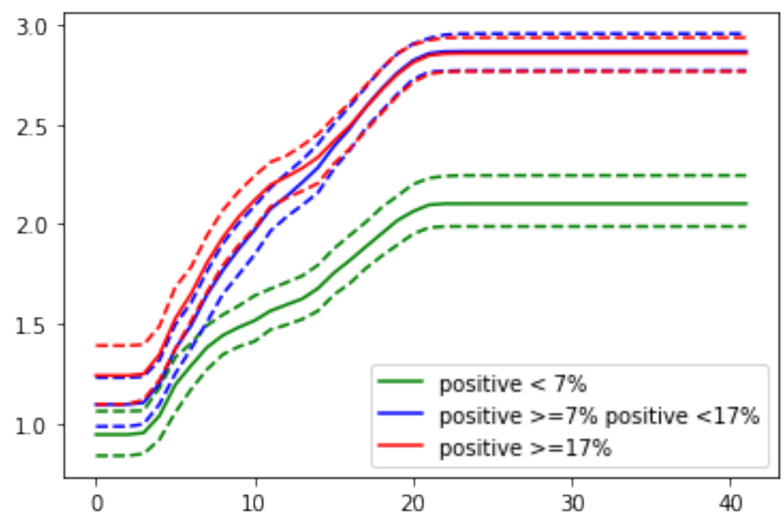

Supplement: Supplementary file 1 [file jcm-09-01904-s001.zip › sup/5_sample_script.ipynb.pdf]
